# Proprotein Convertase Subtilisin/Kexin Type 9 (PCSK9) Inhibitors as Adjunct Therapy to Statins: A New Frontier in Cardiovascular Risk Reduction

**DOI:** 10.7759/cureus.71365

**Published:** 2024-10-13

**Authors:** Tasnia Hossain Lamia, Prince Shah-Riar, Mousumi Khanam, Farzana Khair, Anahita Sadat, Maksuda Khan Tania, Siddiqi M Haque, Shaila S Saaki, Aysha Ferdausi, Sadia Afrin Naurin, Maliha Tabassum, Riffat E. Tasnim Rahie, Rashedul Hasan

**Affiliations:** 1 Internal Medicine, Cox's Bazar Medical College, Cox's Bazar, BGD; 2 Internal Medicine, Doctors Hospital at Renaissance (DHR) Health, Edinburg, USA; 3 Internal Medicine, Dhaka Medical College and Hospital, Dhaka, BGD; 4 Internal Medicine, Bangladesh Medical College, Dhaka, BGD; 5 Internal Medicine, Ibrahim Medical College and Birdem General Hospital, Dhaka, BGD; 6 Internal Medicine, University of California, Riverside School of Medicine, Riverside, USA; 7 Internal Medicine, Ibrahim Medical College, Dhaka, BGD; 8 Internal Medicine, Holy Family Red Crescent Medical College, Dhaka, BGD; 9 Internal Medicine, Desert Valley Hospital, Victorville, USA

**Keywords:** 3-hydroxy-methylglutaryl-coenzyme, alirocumab, cardiovascular disease, cardiovascular mortality, cholesterol, dyslipidemia, evolocumab, ldl, pcsk9, statin

## Abstract

Lowering low-density lipoprotein cholesterol (LDL-C) plasma levels is crucial for the prevention of primary and secondary cardiovascular diseases (CVDs). Many patients struggle to obtain goal LDL-C levels, despite the availability of several lipid-lowering medications, because of limited efficaciousness and unfavorable side effects. Proprotein convertase subtilisin/kexin type 9 (PCSK9) targeting has drawn interest recently as a novel approach to further lower cardiovascular (CV) risk. The number of receptors accessible to remove LDL-C from the bloodstream is reduced when PCSK9 attaches to LDL-C receptors and directs them toward lysosomal destruction. LDL receptor activity is increased by PCSK9 inhibition, which attracts therapeutic intervention. Despite concurrent statin therapy, phase 3 clinical trials have demonstrated encouraging outcomes with monoclonal antibodies against PCSK9, such as evolocumab and alirocumab, resulting in significant reductions in LDL-C levels. This study intends to investigate recent advancements in the field to evaluate PCSK9 inhibitors' safety, effectiveness, and potential for preventing CVD. The investigation will also review potential future paths and wider effects of using PCSK9 inhibitors in therapeutic settings.

## Introduction and background

Cardiovascular disorders (CVDs) impose a significant global burden on both patients and society [1]. Various genetic, epidemiological, and clinical studies strongly support the association of plasma cholesterol, particularly low-density lipoprotein cholesterol (LDL-C), as a primary factor in the initiation and advancement of CVDs [2]. Statins, known as 3-hydroxy-3-methylglutaryl-coenzyme A (HMG-CoA) reductase inhibitors, have been the most common therapy for dyslipidemia for many years [3]. However, regardless of statin drug intolerance, high-risk patients continue to experience potential adverse outcomes, such as statin-associated myopathy [4]. The Food and Drug Administration (FDA) approved two novel drugs, evolocumab and alirocumab, in 2015 due to their significant efficacy in reducing LDL-C [3].

It is possible for alirocumab to lower LDL-C levels by as much as 72.4%. Proprotein convertase subtilisin/kexin type 9 (PCSK9) inhibitors can also lower total cholesterol, non-high-density lipoprotein cholesterol (non-HDL-C), apolipoprotein, lipoprotein(a), and triglycerides (TG) [5]. In addition, it considerably elevates HDL-C and apolipoprotein A-1 levels. When evolocumab is used with statins, it lowers LDL-C levels by up to 60% [6]. This significant reduction can contribute to a decreased probability of CV events in patients with hypercholesterolemia, making PCSK9 inhibitors an essential option for high cholesterol management [7].

In recent years, the incidence and prevalence of CVDs have significantly increased worldwide. In the United States, about 695,000 people die from CVDs each year. This cause accounts for one out of every five fatalities. An estimated 16.3 million Americans aged 20 years and older are affected by CVDs, which is 48.6% of the total population [8]. PCSK9 inhibitors represent a significant breakthrough in CVD treatment, particularly for individuals with high cholesterol levels who are unresponsive to traditional therapies.

As research continues, it promises to further improve CV outcomes and expand the therapeutic options available to patients. This review analyzes the current application of PCSK9 inhibitors in clinical practice and explores how recent and emerging evidence may expand its use in the future [3].

This systematic review aims to evaluate the effectiveness of PCSK9 inhibitors in combination with statins, rather than with statins alone, to lower the risk of CVDs. People who experience intolerance to statins may consider PCSK9 inhibitors as an alternative treatment.

## Review

Methodology

We included any prospective, retrospective, clinical, and preclinical trials meeting our inclusion criteria in the review. Subsequently, the selected studies were assessed based on the inclusion criteria: publication within the last 10 years (February 1, 2014-February 1, 2024), involving adult participants (aged 40 years or older with hyperlipidemia and coronary artery disease), written in English, and available as full-text articles. We conducted a comprehensive literature search using PubMed (using Medline), focusing on PCSK9i monoclonal antibodies, statins, CVDs, mortality benefits, hypercholesterolemia, atherosclerosis, and secondary prevention. A total of 2,070 articles were retrieved that were relevant to our initial keywords from the online database. Eight independent reviewers then screened the titles and abstracts and excluded the duplicate 1,928 non-relevant studies. In the end, full-text screening of the remaining 142 studies was completed to check for eligibility. Subsequently, the studies were subcategorized according to their chronological number. An additional four reviewers resolved any controversies during the screening steps.

**Figure 1:**
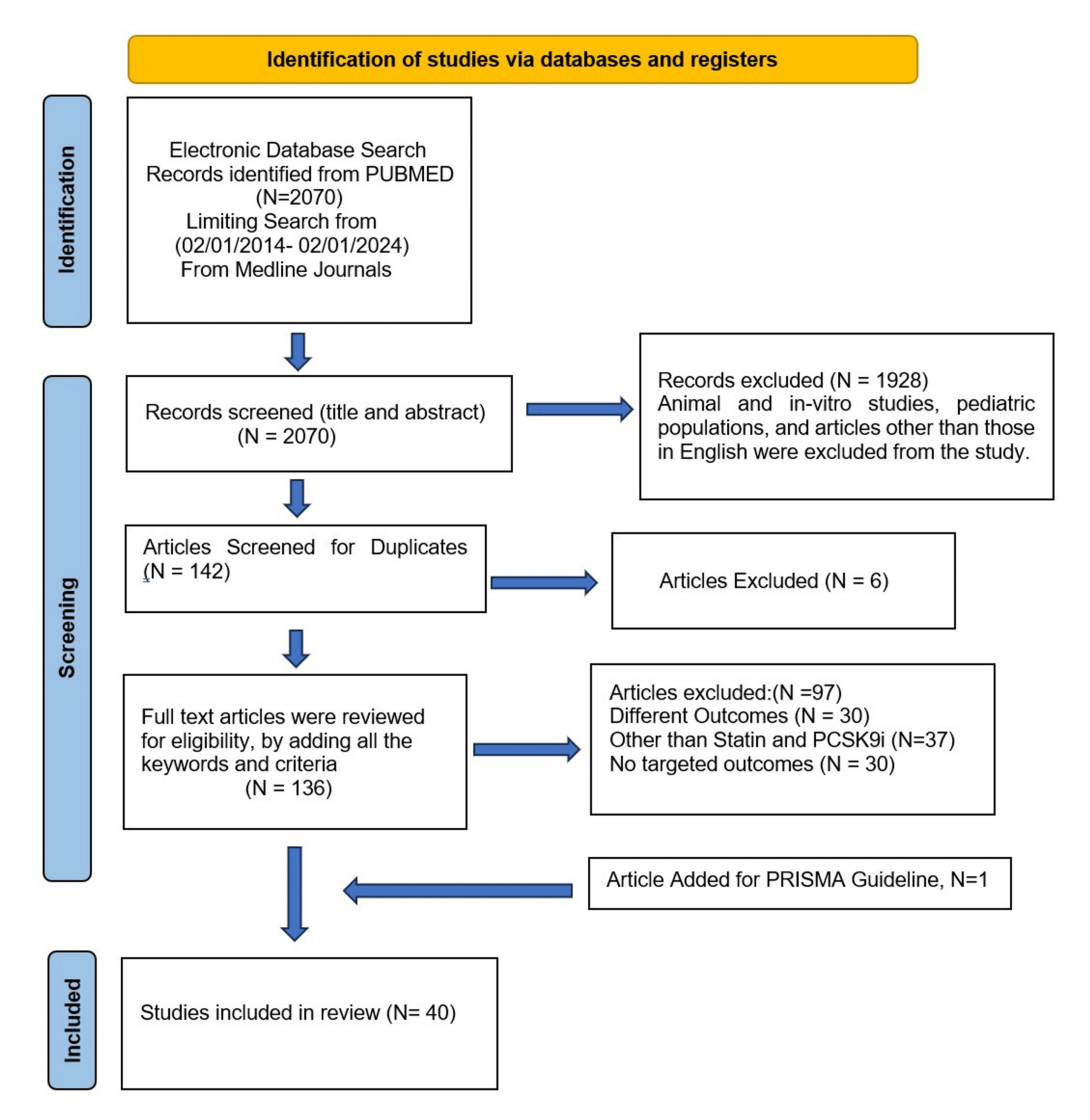
PRISMA Diagram of included articles Image credit: Dr. Prince Shah-Riar PCSK9i: Proprotein convertase subtilisin/kexin type 9 inhibitors; PRISMA: Preferred Reporting Items for Systematic Reviews and Meta-Analyses

Finally, a total of 40 studies (N = 40) were selected as eligible for review (Figure 1). All the articles went through extensive deliberations, scanning the relevant information and paraphrasing the essential points for analysis in Google Spreadsheets (Google, Inc., Mountain View, CA, USA) for four months. Any discrepancies were resolved among the authors through thorough discussion. The screening process adhered to the Preferred Reporting Items for Systematic Reviews and Meta-Analyses (PRISMA) 2020 guidelines to ensure no duplication [9].

PCSK-9 Inhibitors Mechanism

The discovery of PCSK-9 in 2003 revolutionized our understanding of cholesterol metabolism. It paved the way for the development of PCSK-9 inhibitors as a novel class of lipid-lowering drugs. PCSK-9 inhibitors bind to PCSK-9 molecules, thereby preventing their interaction with LDL receptors. This inhibition leads to increased uptake of LDL-C from the bloodstream, resulting in reduced LDL-C levels [10-12]. 

Three monoclonal antibodies - alirocumab, evolocumab, and bococizumab - have demonstrated efficacy in inhibiting PCSK-9 and reducing LDL-C levels. Alirocumab and evolocumab have received FDA approval and are primarily used in treating autosomal familial hypercholesterolemia and statin intolerance. These medications can be used alone or in combination with statins and ezetimibe to achieve therapeutic goals as per guidelines from the American Heart Association (AHA) and the European Society of Cardiology (ESC) [1]. 

**Figure 2:**
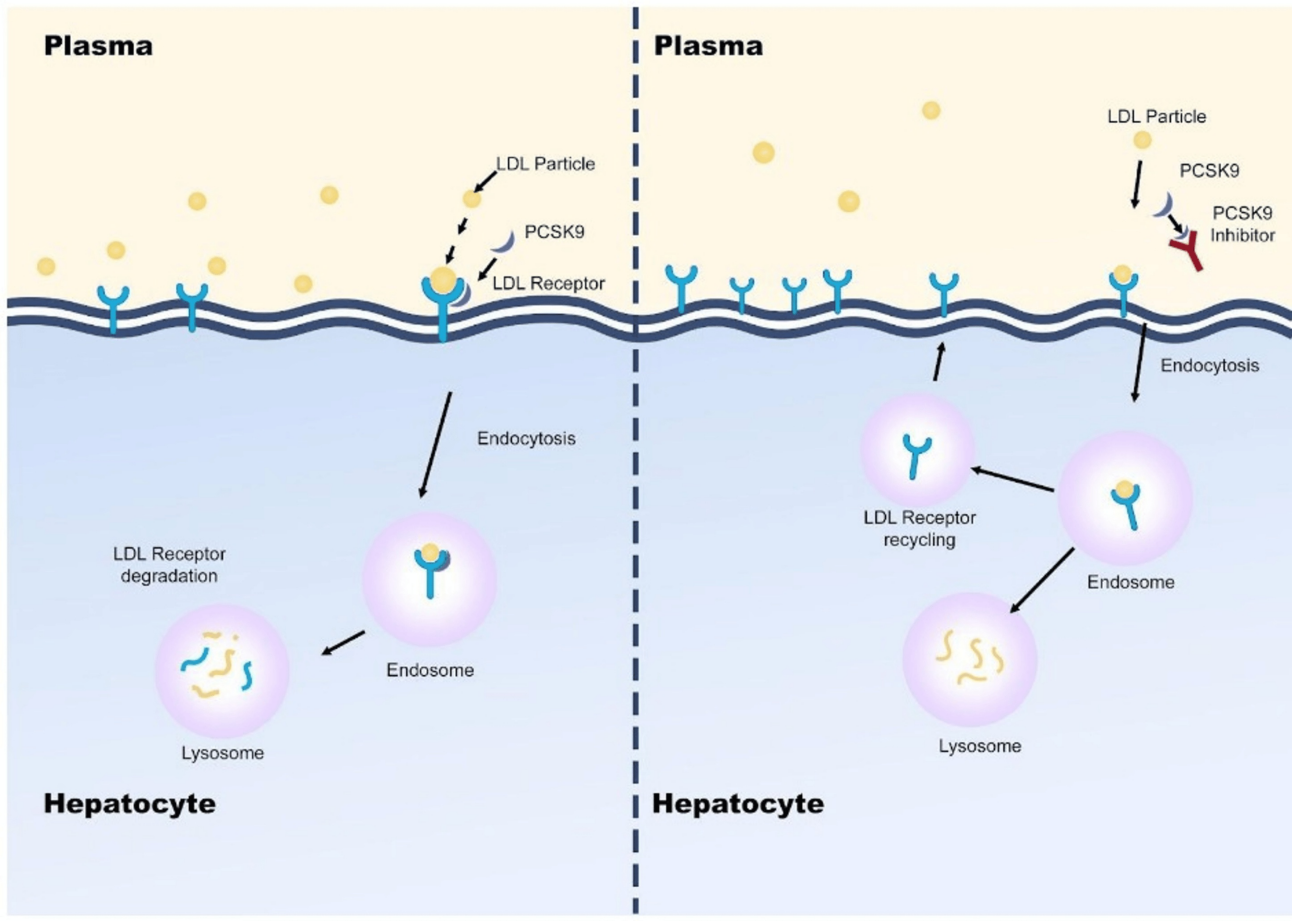
Mechanism of action of PCSK-9 inhibitor on reducing LDL cholesterol Image credit: Dr. Aysha Ferdausi LDL: Low-density lipoprotein; PCSK-9: Proprotein convertase subtilisin/kexin type 9

However, PCSK-9 inhibitors have demonstrated pleiotropic effects beyond their lipid-lowering effects, including anti-atherosclerotic, anti-aggregation, anticoagulant, antineoplastic, and antimicrobial activities. These additional effects make PCSK-9 inhibitors increasingly valuable, especially when standard lipid-lowering therapies fail (Figure 2) [2].

PCSK9 Inhibitors CVD Mortality Benefits

In the FOURIER (Further Cardiovascular Outcomes Research with PCSK9 Inhibition in Subjects with Elevated Risk) trial, patients received either evolocumab or placebo, and evolocumab reduced the risk of CV events such as CV death, myocardial infarction, stroke, or coronary revascularization, but there was no difference in mortality. Evolocumab also demonstrated a decrease in significant adverse limb events compared to placebo while patients were receiving statin treatment. The findings from the FOURIER trial demonstrate a significant reduction in CV events with evolocumab; however, the lack of difference in mortality raises questions about the strength of the causal relationship between LDL-C lowering and overall survival. While the reduction in non-fatal events, such as myocardial infarction and stroke, suggests a clear benefit of evolocumab, the absence of a mortality benefit implies that LDL-C reduction may not be directly causative of improved survival in the short-to-intermediate term [13]. Longer follow-up or additional studies might be needed to clarify whether a causal link exists between prolonged LDL-C reduction and overall mortality.

In the ODYSSEY OUTCOMES trial, alirocumab significantly decreased significant CV events and all-cause mortality. These benefits were related to reducing both LDL-C and lipoprotein(a) [3]. In the OSLER (Open-Label Study of Long-term Evaluation Against LDL-C) trial, evolocumab reduced plasma LDL-C concentration by 61% and CV adverse events at one year, from 2.18% in the standard therapy group to 0.95% [7].

There is evidence that alirocumab, when added in the background of statin therapy, reduces all causes of death after acute coronary syndrome (ACS) in patients with higher levels of atherogenic lipoproteins. More significant treatment benefit occurs beyond the first year, with longer follow-up and higher baseline LDL-C values (>100 mg/dL). A lower risk of subsequent death is associated with the achievement of an LDL-C value <30 mg/dL. This indicates that prolongation of life may be achievable with long-term treatment with alirocumab [14].

A pre-specified analysis of the ODYSSEY OUTCOMES trial showed that, in patients with recent ACS, alirocumab significantly reduced the relative risk of major peripheral artery disease (PAD) events by more than 30%. This risk reduction was most pronounced in those with a prior history of PAD, consistent with the observed effects of evolocumab on PAD events in patients with chronic atherosclerotic CVDs. The findings also suggested that PCSK9 inhibitors could reduce the risk of venous thromboembolism (VTE) when combined with intensive statin therapy in certain patients. However, the analysis is limited due to the short follow-up (2.8 years), which limits long-term conclusions, and the results may not be fully generalizable to patients without recent ACS. Additionally, the high cost of PCSK9 inhibitors raises concerns about accessibility in routine practice. Uncertainty remains around the optimal LDL-C targets and long-term safety, particularly regarding the potential adverse effects of very low LDL-C levels. Addressing these limitations provides a balanced view of the trial’s implications for clinical practice [15].

Statin Mechanism

Statins, widely recognized as HMG-CoA reductase inhibitors, are lauded for their ability to lower cholesterol. They do this by targeting and competitively inhibiting the HMG-CoA reductase enzyme, which is central to the mevalonate pathway responsible for cholesterol and isoprenoid production. This inhibition curtails the production of mevalonate, leading to a decrease in the liver's internal production of cholesterol. As a result, the liver responds by increasing the number of LDL receptors on its surface, facilitating the removal of LDL-C from the bloodstream and thus lowering blood cholesterol levels [11,16]. Beyond their primary cholesterol-lowering capability, statins also exhibit pleiotropic effects, including enhancing endothelial function, stabilizing atherosclerotic plaques, and exerting anti-inflammatory properties. Statins initiate changes within the atherosclerotic plaques, slowing their progression and conferring greater stability to these structures, thereby mitigating the risk of myocardial infarctions and strokes. It is the intricate alterations within the plaque's cellular structure - modifying smooth muscle cells, adjusting the surrounding matrix, and diminishing enzyme activities that degrade the extracellular matrix, such as those of matrix metalloproteinases - that underpin this stabilizing effect [17,18]. A pivotal anti-inflammatory effect of statins is evidenced by their ability to lower levels of C-reactive protein (CRP), an inflammation marker, which is correlated with a reduced occurrence of CV events. This correlation suggests that statins provide CV benefits that extend beyond cholesterol management. Their anti-inflammatory impact may be attributed to the inhibition of pro-inflammatory transcription factors, a shift in the balance of T-cell differentiation, and an influence on the interactions between leukocytes and endothelial cells [17,19]. While statins’ cholesterol-lowering prowess is firmly established and directly associated with decreased CV incidents, their pleiotropic actions are thought to provide supplemental therapeutic advantages. These encompass not just the enhancement of endothelial functions but also the attenuation of inflammatory processes. Together, these factors potentially play a significant role in the comprehensive CV protection offered by statin therapy [16-18].

Mortality Benefits of Statin

Extensive research affirms the efficacy and safety of statins in preventing CVDs [18,20]. They primarily target LDL-C, a key contributor to coronary heart disease (CHD), as evidenced by trials such as the West of Scotland Coronary Prevention Study (WOSCOPS) and Air Force/Texas Coronary Atherosclerosis Prevention Study (AFCAPS/Tex-CAPs) [18]. According to findings from the JUPITER (Justification for the Use of Statins in Prevention: An Intervention Trial Evaluating Rosuvastatin) trial, administering a daily dose of 20 mg of rosuvastatin resulted in a substantial reduction of 44% in the occurrence of the initial major CV events, a significant 54% decrease in all instances of myocardial infarction, and an overall 20% reduction in mortality rates [18]. As a result, regardless of the initial LDL-C levels, early initiation of statin therapy is recommended for all ACS patients [19]. Statins offer benefits beyond cholesterol reduction, including decreased hospitalizations, heart failure prevention, plaque stabilization, mortality benefits for patients with PAD, and enhanced myocardial function [20]. These findings assert the mortality benefits of statins in the comprehensive management of CVDs.

Limitations of Statin Therapy

Extensive research shows that statin therapy has clear benefits. Despite this, several uncertainties exist regarding its optimal utilization, particularly in specific patient groups. Elderly individuals are often excluded from studies, as clinicians raise concerns about their safety and efficacy. Additionally, Asians face challenges due to reduced tolerance to high statin doses [21]. Despite being effective, many patients fail to achieve recommended LDL-C levels and, hence, experience recurrent CV events. Many people discontinue statin treatment due to intolerance, which commonly manifests as muscle-related symptoms. Furthermore, statin use is associated with an increased risk of new-onset diabetes. Although supplementing statin therapy with lipid-lowering agents like ezetimibe has shown some benefit in reducing CV events, the incremental benefit of LDL-C reduction beyond statins is limited [21]. Novel therapies targeting PCSK9 have been introduced, showing substantial LDL-C reductions beyond statins and offering success in achieving previously unattainable LDL-C levels. The need for new agents with greater LDL-C-lowering effects is evidenced by the limited efficacy of current add-on therapies, such as fibrates, niacin, and statin therapies [22,23]. In summary, ongoing research into lipid-lowering strategies, including novel agents like PCSK9 inhibitors, has started to show success in addressing unresolved challenges and improving CV outcomes [24]. 

Benefits of Using PCSK9 Inhibitors Along With Statins

Clinicians managing hypercholesterolemia often treat patients with high CV risk who remain inadequately controlled despite maximum tolerated statin therapy. Major trials, such as FOURIER and ODYSSEY OUTCOMES, have demonstrated that PCSK9 inhibitors, like evolocumab and alirocumab, provide additional reductions in LDL-C levels and CV risks, particularly benefiting high-risk populations, including those with diabetes and PAD. Sub-analyses from these trials highlight that patients with PAD, diabetes, or recent myocardial infarction exhibit the highest risk for major adverse CV events (MACEs) and mortality. For instance, in the ODYSSEY OUTCOMES trial, patients with diabetes experienced a substantial reduction in MACEs when treated with PCSK9 inhibitors in combination with statins, demonstrating notable absolute risk reductions [25-28].

These findings have contributed to updates in cholesterol guidelines, which now emphasize lower LDL-C targets, particularly for high-risk groups, like individuals with type 2 diabetes. The 2018 AHA/American College of Cardiology (ACC) guidelines, for example, recommend more aggressive LDL-C lowering in patients at very high risk, targeting levels as low as possible to prevent recurrent CV events.

Initially, there were concerns about the high cost and perceived value of the PCSK9 antibody class, which might lead to limited use in practice. Over time, prices have reduced, and better patient targeting has improved access to the treatments. Studies comparing them to placebo showed significant reductions in hospitalizations and deaths, along with more days spent out of the hospital. This highlights both the clinical and economic benefits of the therapy, which is now considered cost-effective based on available data. This has led to constrained recommendations for PCSK9 inhibitor usage, focusing primarily on patients who gain the most clinical benefit. By considering these updates and expanding on the impact of cost-effectiveness, clinicians can better tailor treatment strategies for very high-risk or high-risk patients, emphasizing the need for aggressive LDL-C lowering in secondary prevention [24].

Combined Statin and PCSK9 Inhibitor Effects on Mortality

The combination of statins and PCSK9 inhibitors represents a highly effective approach for managing dyslipidemia and reducing CV risks [28-30]. At the molecular level, statins work by inhibiting HMG-CoA reductase, reducing cholesterol synthesis, while PCSK9 inhibitors prevent the degradation of LDL receptors, enhancing LDL clearance from the bloodstream. This synergistic pharmacological action amplifies the reduction of LDL-C levels, resulting in significantly improved CV outcomes [31,32].

For example, adding alirocumab to atorvastatin has shown significantly greater reductions in LDL-C compared to alternative therapies like ezetimibe or higher doses of statins. Alirocumab lowered LDL-C levels more effectively, with patients achieving LDL-C targets at a much higher rate [33]. Clinical data also suggest potent reductions in LDL-C and atherosclerosis when combining these therapies, with one study showing LDL-C dropping from 2.38 to 0.64 mmol/L and a reduction in stenosis from 74.2% to 65.5% after 12 weeks [34].

In the ODYSSEY OUTCOMES trial, adding PCSK9 inhibitors to maximally tolerated statins significantly decreased CV events, highlighting the powerful synergy between these therapies in high-risk populations [35]. However, adherence to statin therapy can be a challenge after initiating PCSK9 inhibitors. One study reported that 16% to 20% of patients discontinue statin use within a year of starting PCSK9 inhibitors, which could compromise the overall clinical benefits [36].

This review explores the tolerability, efficacy, and mortality implications of combined statin and PCSK9 inhibitor therapy, emphasizing the importance of maintaining adherence to both medications. Understanding the synergistic effects at the molecular and pharmacological levels is key to optimizing treatment strategies, improving patient outcomes, and reducing mortality in individuals at heightened CV risk. Through this analysis, we aim to inform strategies for better-managing heart health and reducing mortality in at-risk populations.

Adverse Effects of Statin

Many patients taking statins do not achieve the optimal LDL-C levels of <70 mg/dL (1.8 mmol/L) or 55 mg/dL (1.4 mmol/L), as recommended by current consensus guidelines [23]. Additionally, some patients experience recurrent ischemic events despite receiving maximally tolerated statin therapy.

A common complaint among patients on statin therapy involves muscle-related symptoms, such as myalgia (muscle pain), which affects 1-10% of patients. These symptoms often lead to treatment interruption or discontinuation. In rare, severe cases, statin use can lead to rhabdomyolysis, with an incidence rate of less than 0.1% [15]. Furthermore, statin therapy has been associated with an increased risk of new-onset type 2 diabetes mellitus, particularly in patients predisposed to diabetes [3]. However, the CV benefits of statin therapy generally outweigh this risk, as shown in several large-scale trials, including the JUPITER trial, where the reduction in CV events surpassed the increase in diabetes risk [18].

Statins might interfere with liver function, but severe liver damage is rare. The management of elevated liver enzymes in patients taking statins typically involves either reducing the dose or switching to another statin [17]. For patients experiencing elevated liver enzymes, management strategies typically include dose reduction or switching to a different statin with a better safety profile.

There has been some concern over statin-associated cognitive impairments, such as memory loss, behavioral changes, and concentration difficulties. However, data on this topic are mixed. While some studies have suggested a potential link, such as in the PROSPER (Prospective Study of Pravastatin in the Elderly at Risk) trial, others, including a review from the FDA, have found no consistent evidence to support a direct connection between statin use and cognitive decline. It is important to note that cognitive side effects remain rare and should be weighed against the significant CV benefits of statin therapy. Further research is needed to clarify these associations.

In addition, statins are occasionally linked to gastrointestinal side effects, such as nausea and changes in bowel habits, though these are infrequent. Managing these side effects typically involves adjusting the dosage or switching to a statin with fewer gastrointestinal effects, such as pravastatin.

Adverse Effects of PCSK9 Inhibitors

PCSK9 inhibitors have demonstrated significant efficacy in lowering LDL-C levels, although some adverse effects have been reported. The most common side effects observed in studies include influenza-like illness (27.9%), nasopharyngitis (16.2%), and myalgia (10.3%) [7]. Approximately 33% of patients experienced more than one injection site reaction, such as injection site hematoma [37].

Although myalgia was a key reason for discontinuation, the overall discontinuation rate was low. A specific study should be cited here to provide a clearer percentage of discontinuation. It is important to note that the frequency and severity of these adverse effects were generally mild, and most resolved with continued therapy [38].

Injection site reactions, reported in a notable proportion of patients, should be contextualized by comparing them to reactions seen with other injectable medications. Biologics often carry a higher risk of injection site reactions compared to traditional therapies, and understanding this comparison can help clinicians manage patient expectations. In contrast, Myers et al. reported that individuals with access to PCSK9 inhibitors experienced higher CV events compared to the general population, though the underlying reasons for this discrepancy require further investigation [39].

However, most adverse effects were mild and self-limiting, with many resolving during follow-up without requiring discontinuation (Table 1) [40].

**Table 1:** Tabulation of all articles CVD: Cardiovascular disease; ASCVD: Atherosclerotic cardiovascular disease; NSTEMI: Non-ST elevation myocardial infarction; ACS: Acute coronary syndrome; DM: Diabetes mellitus; CKD: Chronic kidney disease; LDL: Low density lipoprotein; PAD: Peripheral artery disease

Authors	Year	Country	Sample size	Duration	Drug	Study design	Key findings	Results	Comorbidities
van den Heuvel et al. [1]	2016	Netherlands	91 medicines	2015	Evolocumab, alirocumab, sacubitril, valsartan, mepolizumab	Narrative review	PCSK9 inhibitors (evolocumab, alirocumab) for hypercholesterolemia, neprilysin inhibitors for heart failure, IL-5 inhibitors for asthma.	Alirocumab and evolocumab are approved for the treatment of primary hypercholesterolemia (both non-familial and heterozygous familial) or mixed dyslipidemia in adults. These drugs are used as an adjunct to diet either: (a) in combination with a statin, or with a statin and other lipid-lowering therapies, for patients who cannot reach LDL-C targets with the maximum tolerated statin dose; or (b) as monotherapy or in combination with other lipid-lowering therapies for patients who are statin-intolerant or for whom statins are contraindicated.	Hypercholesterolemia, asthma, cancer
Trankle al al. [2]	2019	Virginia, USA	20	14 Days	Alirocumab, rosuvastatin, atorvastatin	Randomized controlled trial (RCT) double-blinded	The study evaluated the efficacy of alirocumab in reducing LDL-C levels and improving outcomes in AMI patients.	PCSK9 inhibitor in a hospitalized population being acutely managed for AMI. Within only 72 hours, a statistically significant reduction of LDL-C was found for those who received treatment, a reduction which was further enhanced at day 14. The observation of decreased LDL-C and free PCSK9 in the setting of increased total PCSK9 confirms that bound PCSK9 accumulates in the plasma circulation but remains physiologically inactive.	Acute coronary syndrome
Cho and Hong [3]	2020	Korea	27,564	2.2 years	Evolocumab, alirocumab, ezetimibe	Review article	PCSK9 inhibitors, such as alirocumab and evolocumab, significantly reduce LDL-C levels and lower cardiovascular event risk.	Current guidelines for the management of dyslipidemia recommend the use of PCSK9 inhibitors in patients at high or very high risk of future cardiovascular events, in whom LDL-C targets are not achieved with maximally tolerated statins and ezetimibe, and in those who are statin intolerant.	ASCVD
Al-Mohaissen et al. [4]	2016	Riyad, Saudi Arabia	NA	NA	Ezetimibe resin, niacin bile acid sequestrants PCSK9 inhibitor	Narrative review	Summarizes the incidence, mechanisms, and management of SMAEs, such as myalgia, myopathy, and rhabdomyolysis.	The patient is within 20% of their clinical LDL-C goal, then ezetimibe is preferred; in contrast, for patients who need a >20% reduction in their LDL-C levels, the choice is between multiple standard agents added sequentially (e.g., ezetimibe, a bile acid sequestrant, and niacin), PCSK9 inhibitors, lomitapide or mipomersen (for patients with homozygous familial hypercholesterolemia), apheresis (if available) or complex polypharmacy.	ASCVD hyperlipidemia
Glavinovic et al. [5]	2022	Quebec, Canada	NA	NA	Statins	Review article	ApoB is a superior cardiovascular risk marker because it accounts for all lipoprotein particles that can cause atherosclerosis.	ApoB measurement provides a more accurate understanding of residual cardiovascular risk, as it reflects the number of atherogenic particles rather than just the cholesterol mass within those particles. This allows for more precise risk stratification and can guide adjustments in therapeutic management, particularly in patients with discordant LDL-C and ApoB levels, leading to more targeted interventions.	Dysbetalipoproteinemia, hypertriglyceridemia
Ruscica et al. [6]	2020	Italy	NA	NA	Methotrexate, canakimuab, colchicine, ezetimibe	Narrative review	Highlights the central role of inflammation in atherosclerosis and cardiovascular diseases and reviews anti-inflammatory treatments to reduce cardiovascular risk.	Dietary components, such as unsaturated fatty acids and plant-derived nutraceuticals, show potential in reducing inflammation and cardiovascular risk. However, their efficacy in reducing major cardiovascular events is less established compared to pharmacotherapy like statins and PCSK9 inhibitors, which have proven benefits in lowering LDL-C and preventing adverse cardiovascular outcomes.	CVD with inflammation
Latimer et al. [7]	2016	UK	405	42 weeks	Evolocumab, alirocumab	Review article	PCSK9 inhibitors significantly reduce LDL-C levels and lower cardiovascular events in high-risk patients.	LDL-C levels of up to 70% may be achieved with PCSK9 inhibition.	CVD hypercholesteremia
Benjamin et al. [8]	2017	USA	12878	2010-2020	Statins (for lowering LDL-C), antihypertensive agents (like ACE inhibitors or ARBs), and anticoagulants (e.g., aspirin)	Comprehensive report providing updated statistics	Highlights the prevalence, mortality, and risk factors associated with heart disease and stroke and underscores the need for improved prevention strategies.	An estimated 92.1 million US adults have at least 1 type of CVD. By 2030, 43.9% of the US adult population is projected to have some form of CVD. From 2004 to 2014, death rates attributable to CVD declined 25.3%. The actual number of CVD deaths decreased by 6.7%. Approximately 795,000 strokes occur in the United States each year. On average, every 40 seconds, someone in the US has a stroke, and on average, every 4 minutes, someone dies of a stroke.	CVD, stroke, smoking, high blood cholesterol, high BP, DM
Schmidt et al. [10]	2017	UK, London	67,237 participants (median age 61 years; range 52 to 64 years)	Short-term (24 weeks), medium-term (one year), and long-term (five years)	Ezetimibe, statins, alirocumab, evolocumab, bococizumab	A systematic review conducted by the Cochrane	This article summarizes clinical trials showing that PCSK9 monoclonal antibodies, such as alirocumab and evolocumab, significantly reduce LDL-C levels and the risk of cardiovascular events.	PCSK9 inhibitors decreased the risk of CVD events, with a risk difference (RD) of 0.91% (odds ratio (OR) of 0.86, 95% CI 0.80 to 0.92; eight studies; 59,294 participants; GRADE: moderate). Compared with ezetimibe and statins, PCSK9 inhibitors appeared to have a more substantial protective effect on CVD risk, although with considerable uncertainty (RD 1.06%, OR 0.45, 95% CI 0.27 to 0.75).	CVD, DM, cancer
Ciccarelli et al. [11]	2018	Naples, Italy	NA	NA	Ezetimibe, simvastatin, evolocumab, alirocumab, bococizumab	Review article	Discusses the effectiveness of PCSK9 monoclonal antibodies in significantly lowering LDL-C levels in high-risk cardiovascular patients and reducing cardiovascular events.	ASCVD (55 years of age for males or <65 years of age for females) in which an LDL-C goal of less than 55 mg/dL is recommended. The 30 mg/dL goal, as indicated in most of the PCSK9 inhibitors clinical trials, is desirable.	ASCVD with DM, CKD
Khatib et al. [12]	2022	UK	NA	NA	PCSK9 inhibitors such as alirocumab and evolocumab	A descriptive study	A centralized, multidisciplinary approach improves outcomes, adherence, and cost-effectiveness for high-risk cardiovascular patients.	-	Cardiovascular disease
Sabatine et al. [13]	2017	International, across 49 countries	27654	Median follow-up of 2.2 years	Evolocumab	Randomized controlled trial (FOURIER trial)	Evolocumab significantly reduced LDL cholesterol levels and was associated with a lower risk of major cardiovascular events, including myocardial infarction, stroke, and coronary revascularization. The greatest benefit was seen in patients with the lowest LDL cholesterol levels.	LDL Cholesterol Reduction with Evolocumab: 59% (from 92 mg/dL to 30 mg/dL) vs. placebo (P<0.001) Primary End Point Risk Reduction: Evolocumab (9.8%) vs. placebo (11.3%); Hazard Ratio (HR) 0.85 (95% CI, 0.79 to 0.92) (p<0.001) Key Secondary End Point Risk Reduction: Evolocumab (5.9%) vs. placebo (7.4%); HR 0.80 (95% CI, 0.73 to 0.88) (p<0.001).	Atherosclerotic cardiovascular disease and LDL cholesterol levels of 70 mg
Steg et al. [14]	2019	Europe (51%), Canada and the United States (approximately 75%), Latin America (14%), and Asia (12%)	18924	Average 2.8 years (2.3-3.4 years)	Alirocumab	Clinical trial	Alirocumab significantly reduced all-cause and cardiovascular mortality, especially in patients with high LDL levels despite statin therapy.	Premature treatment discontinuation other than death occurred due to low LDL levels in 1343 patients and with placebo in 1496 patients.	Cardiovascular disease
Schwartz et al. [15]	2020	Europe, Asia, North America, Latin America	18924	2.8 years	Statin, alirocumab, placebo	Post-hoc analysis of a clinical trial	PAD in ACS patients was associated with increased mortality and major cardiovascular events, emphasizing aggressive risk management.	In patients with recent acute coronary syndrome receiving statin therapy, alirocumab has significantly reduced lipoprotein levels compared to placebo.	Acute coronary syndrome
Koskinas et al. [16]	2021	NA	NA	NA	NA	Review article	Intensive lipid-lowering therapy (statins, PCSK9 inhibitors) reduces major adverse cardiovascular events in PCI patients. The duration and intensity of therapy are essential.	NA	Coronary artery disease
Yu and Liao [17]	2022	NA	3086	NA	NA	Review article	Statins reduce LDL cholesterol and have anti-inflammatory, vascular protective properties, stabilizing atherosclerotic plaques.	Statins reduce cardiovascular disease risk not only by lowering cholesterol but also by improving vascular function and reducing inflammation.	Coronary artery disease
Rossini et al. [18]	2022	Italy	534	8 weeks	Rosuvastatin, atorvastatin, simvastatin	Review article	Factors like diabetes and kidney disease affect the efficacy of statins. Personalizing treatment based on patient characteristics is essential to minimize risks.	Statin has shown effectiveness in CVD disease with avoidance in some diseases.	CKD, liver disease, cancer, muscle disorder
Kowara and Cudnoch-Jedrzejewska [19]	2021	Poland	697	3 years	Statin, alirocumab, ezetimibe	Review article	Inflammation, lipid levels, and other factors influence plaque stability. PCSK9 inhibitors, anti-inflammatory drugs, and novel therapies reduce the risk of plaque rupture.	Discusses five major therapeutic strategies aimed at stabilizing vulnerable atherosclerotic plaques.	Cardiovascular disease
Krähenbühl et al. [20]	2016	NA	NA	NA	Statins, PCSK9 inhibitors, ezetimibe, CETP inhibitors	Review article	PCSK9 inhibitors, ezetimibe, and other emerging lipid-lowering therapies address unmet needs in LDL-C lowering for statin-intolerant or resistant patients.	Statins are insufficient for many patients in LDL-C lowering, alternative or combination therapies should be considered.	Cardiovascular disease
Cannon et al. [21]	2015	England	18144	6 years median followup	Ezetimibe, statin	Randomized controlled trial	Adding ezetimibe to statin therapy resulted in further LDL reduction and a significant reduction in major cardiovascular events compared to statin therapy alone.	Patients with ACS, along with some other comorbid conditions, showed a higher result when ezetimibe-Simvastatin combined.	Hypercholesterolemia
Jakob et al. [22]	2016	Systematic review and meta-analysis of global studies	NA	Studies with varying durations included in the systematic review	Fibrates (e.g., gemfibrozil, fenofibrate)	Systematic review and meta-analysis from Cochrane	Fibrates reduce triglyceride levels, but their effectiveness in reducing cardiovascular events is uncertain. More high-quality trials are needed.	Fibrates may have benefits in reducing lipid levels but do not significantly impact major cardiovascular events in primary prevention.	Hypercholesterolemia
Schandelmaier et al. [23]	2017	North America, Europe, Asia	39195	1968-2015	Niacin, statin	Systematic review and meta-analysis from the Cochrane Database	The review found that while niacin effectively increases HDL cholesterol and reduces triglycerides, there is no significant evidence that niacin reduces cardiovascular mortality or major adverse cardiovascular events in primary or secondary prevention. The study also highlighted concerns regarding the adverse effects associated with niacin therapy.	Niacin does not reduce any other cardiovascular outcomes such as cardiovascular mortality, non‐cardiovascular mortality, fatal myocardial infarctions, non‐fatal myocardial infarction, or fatal or non‐fatal myocardial infarction	Cardiovascular disease
Duprez et al. [24]	2020	Multi-center trials in different regions	NA	NA	Arilocumab, evolocumab, statin	Review article	The article highlights the substantial LDL cholesterol reduction and cardiovascular event risk reduction achieved with PCSK9 inhibitors such as alirocumab and evolocumab. It discusses the benefits observed in primary and secondary prevention settings and examines ongoing studies investigating these treatments' long-term safety and efficacy.	High CV risk patients or in patients with recent ACS, the PCSK9 inhibitors evolocumab and alirocumab achieved additional LDL-C and CV risk reduction beyond that seen with statins	ASCVD, elevated level of LDL
Murphy et al. [25]	2019	Multinational (49 countries)	27564	2017-2019	Evolocumab	A prespecified analysis of the FOURIER trial	The analysis found that evolocumab significantly reduced cardiovascular events (including recurrent events) in patients with atherosclerotic cardiovascular disease. This effect was consistent across subgroups of high-risk populations.	Lipid-lowering therapy with the PCSK9 inhibitor evolocumab improved clinical efficacy, reducing total primary end-point events in patients with stable cardiovascular disease receiving statin therapy.	Myocardial infarction, stroke, and coronary revascularization
Bonaca et al. [26]	2018	Multi-center international study	27564	2.5 years	Evolocumab	A prespecified analysis of the FOURIER trial	The study found that evolocumab significantly reduced LDL-C levels and improved cardiovascular outcomes in patients with PAD, reducing the risk of major adverse cardiovascular events. These findings highlight the benefit of LDL-C lowering therapy in this population.	Evolocumab significantly reduced the primary endpoint consistently in patients with PAD (hazard ratio (HR) 0.79; 95% confidence interval (CI), 0.66-0.94; p=0.0098) and without PAD (HR 0.86; 95% CI, 0.80-0.93; p=0.0003; pinteraction=0.40)	PAD
Schwartz et al. [27]	2018	Multicenter trial	18924	2 years	Alirocumab, statin	Randomized controlled trial (ODYSSEY OUTCOMES trial)	The study found that alirocumab significantly reduced the risk of major adverse cardiovascular events, including myocardial infarction and stroke, in patients who had experienced ACS and had elevated LDL-C despite statin therapy. The greatest benefit was observed in patients with higher baseline LDL levels.	Composite Primary End-Point Event: alirocumab: 9.5% (903 patients); placebo: 11.1% (1,052 patients); hazard ratio: 0.85 (95% CI, 0.78 to 0.93; p < 0.001). Mortality: alirocumab: 3.5% (334 patients); placebo: 4.1% (392 patients); hazard ratio: 0.85 (95% CI, 0.73 to 0.98). Benefit Analysis: Greater benefit of alirocumab observed in patients with baseline LDL-C ≥100 mg/dL.	ACS
Sabatine et al. [28]	2017	International	27564	Median follow-up of 2.2 years	Evolocumab	A prespecified analysis of the FOURIER trial	The study found that evolocumab reduced cardiovascular events in patients with and without diabetes. It did not significantly affect glycemia or increase the risk of new-onset diabetes, making it safe for use in patients with diabetes.	Evolocumab significantly reduced cardiovascular outcomes consistently in patients with and without diabetes at baseline. For the primary composite endpoint, the hazard ratios (HRs) were 0·83 (95% CI 0·75-0·93; p=0·0008) for patients with diabetes and 0·87 (0·79-0·96; p=0·0052) for patients without diabetes (pinteraction=0·60). For the key secondary endpoint, the HRs were 0·82 (0·72-0·93; p=0·0021) for those with diabetes and 0·78 (0·69-0·89; p=0·0002) for those without diabetes (pinteraction=0·65). Evolocumab did not increase the risk of new-onset diabetes in patients without diabetes at baseline (HR 1·05, 0·94-1·17), including in those with prediabetes (HR 1·00, 0·89-1·13).	Diabetes, CAD
Nicholls et al. [29]	2016	North America, Europe, and South Africa	968	78 weeks	Combined	A randomized controlled trial	Evolocumab significantly reduced LDL-C and slowed the progression of coronary atherosclerosis in statin-treated patients. In some patients, it even led to regression of plaque volume, supporting its role in aggressive lipid-lowering therapy.	LDL-C levels: difference: -56.5 mg/dL, 95% CI: -59.7 to -53.4, p < 0.001. Primary Efficacy (PAV): Increased by 0.05% with placebo and decreased by 0.95% with evolocumab (difference: -1.0%, 95% CI: -1.8% to -0.64%, p < o.001). Plaque Regression: Evolocumab induced plaque regression in 64.3% of patients vs. 47.3% with placebo (difference: 17.0%, 95% CI: 10.4% to 23.6%, p < 0.001 for PAV; and 61.5% vs. 48.9% for TAV, difference: 12.5%, 95% CI: 5.9% to 19.2%, p < 0.001).	Angiographic coronary disease
Nicholls et al. [30]	2022	Multicenter, international study	137	52 weeks	Evolocumab, a PCSK9 inhibitor	A prospective clinical trial	Evolocumab significantly reduced coronary plaque burden and induced favorable changes in plaque phenotype, including a reduction in lipid content, suggesting its role in plaque stabilization in patients with a history of myocardial infarction.	LDL-C Levels: Evolocumab group: 28.1 mg/dL vs. Control: 87.2 mg/dL (p < 0.001); Minimum Fibrous Cap Thickness: Evolocumab: +42.7 μm vs. Control: +21.5 μm (p = 0.015); Maximum Lipid Arc: Evolocumab: -57.5° vs. Control: -31.4° (p = 0.04); Percent Atheroma Volume: Evolocumab: -2.29% ± 0.47% vs. Control: -0.61% ± 0.46% (p = 0.009).	NSTEMI
Räber et al. [31]	2022	Multi-center study conducted in Europe	300	52 weeks	Alirocumab (a PCSK9 inhibitor) added to high-intensity statin therapy (atorvastatin or rosuvastatin).	Randomized controlled trial (PACMAN-AMI)	The study demonstrated that adding alirocumab to high-intensity statins resulted in more significant reductions in coronary plaque volume and improved plaque characteristics than statin therapy alone. This suggests enhanced benefits for plaque regression and stabilization in high-risk patients’ post-myocardial infarction.	Percent Atheroma Volume Change (52 weeks): Alirocumab: -2.13% vs. Placebo: -0.92%; Difference: -1.21% (95% CI: -1.78% to -0.65%, p < 0.001). Maximum Lipid Core Burden Index Change (4 mm): Alirocumab: -79.42 vs. Placebo: -37.60; Difference: -41.24 (95% CI: -70.71 to -11.77, p = 0.006). Minimal Fibrous Cap Thickness Change: Alirocumab: 62.67 μm vs. Placebo: 33.19 μm; Difference: 29.65 μm (95% CI: 11.75 to 47.55, p = 0.001).	Acute MI
Ray et al. [32]	2020	United States ORION-11: International (Europe and South Africa)	3178	540 days	Inclisiran	Two phase 3, randomized, double-blind, placebo-controlled trials (ORION-10 and ORION-11)	Inclisiran, a small interfering RNA therapy targeting PCSK9 synthesis, significantly reduced LDL cholesterol levels in patients with atherosclerotic cardiovascular disease or risk equivalents, showing sustained LDL reductions with an infrequent dosing schedule (every six months). The treatment was well tolerated with a safety profile similar to placebo.	Time-Adjusted LDL Cholesterol Reduction: ORION-10: 53.8% (95% CI: 51.3 to 56.2); ORION-11: 49.2% (95% CI: 46.8 to 51.6). Statistical Significance: p < 0.001 vs. placebo.	ASCVD elevated LDL on statin therapy
Stiekema et al. [33]	2019	Netherlands, Canada, and United States	129	2016-2018	Evolocumab	Observational study	The study found that while PCSK9 inhibitors significantly lowered LDL-C levels, patients with elevated lipoprotein(a) continued to exhibit persistent arterial wall inflammation, suggesting that lipoprotein(a) may contribute to residual cardiovascular risk independent of LDL-C.	Evolocumab significantly reduced LDL-C at week 16 (mean (95% CI) percent change treatment difference; total cholesterol and triglycerides were also reduced mean (SD) LDL-C was 1.6 (0.7) mmol/L (60.1 (28.1) mg/dL) at week 16 in the evolocumab group. Evolocumab resulted in a mean (95% CI) percent change treatment difference in Lp(a) vs. placebo of -13.9% (-19.3% to -8.5%; p < 0.001).	Arterial wall inflammation
Bays et al. [34]	2015	Australia, Canada, France, Germany, Italy, Mexico, Spain, the United Kingdom, and the United States	355	2012-2014	Alirocumab, statin, ezetimibe	Randomized controlled trial (ODYSSEY OPTIONS I)	Alirocumab added to atorvastatin resulted in significantly greater reductions in LDL-C compared to other lipid-lowering strategies, including ezetimibe and increasing statin doses. Alirocumab was well tolerated and demonstrated superior LDL-C lowering efficacy.	For patients receiving alirocumab added to background atorvastatin 20 mg and 40 mg, the combined proportion of high and very-high CVD risk patients achieving protocol predefined LDL-C goals of less than 70 mg/dL and less than 100 mg/dL, respectively, at week 24, was greater than 80% when using calculated LDL-C values.	CHD, or type 2 diabetes with target organ damage
Wu et al. [35]	2023	China	49	12 weeks	Atorvastatin, evolocumab	High-resolution MRI study	The study found that PCSK9 inhibition combined with statin therapy led to significant improvements in the reduction of intracranial atherosclerotic plaque volume and stenosis, providing a potential therapeutic strategy for intracranial atherosclerosis.	Forty-nine individuals were studied (PCSK9i group: 26 individuals with 28 abnormal vascular regions; statin group: 23 with 27 regions). The PCSK9i group showed a significant reduction in the normalized wall index (0.83 vs. 0.86, p = 0.028) and stenosis degree (65.5% vs. 74.2%, p = 0.01). Similarly, a greater percentage of individuals with a good response to the efficacy of treatment were treated in the PCSK9i group than in the statin group (75% vs. 44.4%, p = 0.021). The incidence of major vascular events was overall similar between the groups. The treatment options (OR = 8.441, p = 0.01) and prior diabetes (OR = 0.061, p = 0.001) were significantly associated with the efficacy of treatment.	Hypertension, previous ischemic stroke, diabetes
Cupido and Kastelein [36]	2020	Multinational ORION trials	ORION-9: 482 participants; ORION-10: 1,561 participants; ORION-11: 1,617 participants	Follow-up durations of approximately 18 months	Inclisiran	Review article discussing the outcomes and implications of the ORION trials, which evaluated inclisiran, a small interfering RNA therapy, for the treatment of hypercholesterolemia. The article also highlights unanswered questions regarding its long-term effects and broader clinical use.	Inclisiran demonstrated effective and sustained LDL-C reduction with biannual dosing in the ORION trials. However, the authors raise questions about long-term cardiovascular outcomes, safety, and potential effects on broader patient populations, which remain to be fully addressed.	Inclisiran reduced plasma PCSK9-levels with approximately 80% without any indication that this reduction attenuated over the duration of the trials. Moreover, inclisiran also significantly lowered total cholesterol, non-high-density lipoprotein cholesterol (non-HDL-C), apolipoprotein B, and triglycerides and was associated with an 18.6-25.6% reduction in lipoprotein(a) (LP(a)) levels. HDL-C levels increased in the inclisiran groups, but no differences in C-reactive protein (CRP) levels were found.	Cardiovascular disease
Bodapati et al. [37]	2023	NA	41	2010-2013	Evolocumab, alirocumab	Systematic review and meta-analysis evaluating the impact of PCSK9 inhibitors on cardiovascular outcomes, including major adverse cardiovascular events (MACEs) and mortality.	The meta-analysis found that PCSK9 inhibitors significantly reduced the risk of major adverse cardiovascular events (MACEs) and all-cause mortality, particularly in high-risk populations with elevated LDL-C. The review confirmed the efficacy and safety of PCSK9 inhibitors as part of a comprehensive lipid-lowering strategy.	Alirocumab use was linked to a reduced risk of all-cause death compared to control, but not evolocumab. Each of the drugs, evolocumab and alirocumab, significantly reduced the risk of myocardial infarction (MI), coronary revascularization, and ischemic stroke. In comparison to the control therapy, the risk of major detrimental sequelae was significantly reduced by alirocumab therapy in the subgroup analysis of each PCSK9 inhibitor, whereas evolocumab treatment did not demonstrate significant differences (RR = 0.88; 95% CI = 0.72-1.04; evolocumab: RR = 0.99; 95% CI = 0.87-1.11).	Established ASCVD, CHD
Gürgöze et al. [38]	2019	NA	3 different data sources	NA	Evolocumab	Observational study analyzing real-world data on adverse events linked to PCSK9 inhibitors (such as alirocumab and evolocumab) in patients receiving these treatments.	The study identified several common adverse events related to PCSK9 inhibitors, including injection site reactions, influenza-like symptoms, and neurocognitive issues. Despite these events, the overall safety profile was acceptable, with no significant increase in serious adverse events.	In a real-world setting, PCSK9 inhibitors are well tolerated with an overall safety profile comparable to RCTs.	Hypertension, diabetes, CVD
Myers et al. [39]	2019	NA	139036	2015-2017	Alirocumab and evolocumab	Observational study assessing the impact of patient access to prescribed PCSK9 inhibitors on cardiovascular outcomes in real-world settings.	The study found that patients with access to PCSK9 inhibitors had better cardiovascular outcomes, including reduced risk of major adverse cardiovascular events (MACEs), compared to those who were denied access to these treatments. The findings underscore the importance of access to PCSK9 inhibitors for high-risk patients.	Of the 139036 patients prescribed a PCSK9i, exposure cohorts were 32886 (24%) for paid (PD) group, 85370 (61%) for rejected (RJ) group, and 20780 (15%) for abandoned (AB) group. Also, among those prescribed PCSK9is, 88770 (63.8%) had a history of ASCVD before their FAS date and 2889 (2.1%) had a documented diagnosis of FH. Of this latter group, 1944 (1.4%) also had a history of ASCVD before their FAS date. A total of 49321 individuals (35%) had no diagnosis of FH or preFAS ASCVD.	FH, diabetes, ASCVD
Tavori et al. [40]	2015	Oregon, USA	NA	20 years	Evolocumab, bococizumab, alirocumab	The review article summarizes recent findings on the biological mechanisms of PCSK9 and its role in cardiovascular disease, focusing on how PCSK9 inhibition can reduce cardiovascular risk.	The study highlights PCSK9's critical role in regulating LDL-C levels and discusses how PCSK9 inhibitors (such as alirocumab and evolocumab) can significantly reduce LDL levels, providing substantial cardiovascular benefits. The review emphasizes the potential of PCSK9 inhibitors to address unmet needs in lipid management.	LDLR that is defective in LDL uptake can still bind (and be degraded by) PCSK9, but the complete absence of LDLR disallows the benefits of anti-PCSK9 therapy. By extrapolation, these results suggest the following scenarios in patients carrying hypothetical LDLR mutations with defective binding to PCSK9.	Hypercholesterolemia

Limitations

The search was limited to PubMed-indexed journals, which may have excluded relevant studies from other databases, such as EMBASE or the Cochrane Library, potentially impacting the comprehensiveness of the findings. The focus on adults over 40 years of age restricts the applicability of the results to younger populations, who might respond differently to PCSK9 inhibitors due to variations in lipid metabolism and CV risk profiles. Additionally, participants with hyperlipidemia and CAD were included, but those with other CV risk factors, such as diabetes or PAD, were excluded. This exclusion may affect the generalizability of the findings to a broader CV population. Non-English studies were also excluded, which could lead to bias in understanding global treatment outcomes and practice variations in non-English-speaking regions. Only studies published within the past 10 years were included, which may overlook seminal studies that provided foundational insights into the early understanding of PCSK9 inhibitors. Lastly, the exclusion of animal studies limits the inclusion of preclinical data, which could have been valuable in exploring the mechanisms of action of PCSK9 inhibitors, especially in early-stage research.

## Conclusions

The discovery of PCSK9 as a key regulator of LDL receptors has transformed our understanding of cholesterol metabolism, previously thought to be primarily controlled by the SREBP (Sterol Regulatory Element-Binding Protein) pathway. This insight has led to the development of PCSK9 inhibitors, which effectively lower LDL-C levels and may stabilize atherosclerotic plaques, potentially preventing CV events. While initial studies suggest these medications have modest side effects and significant benefits, further research is needed to confirm their long-term impact on cardiovascular health and their anti-inflammatory effects.

Both alirocumab and evolocumab have shown effectiveness in reducing LDL-C, particularly in patients with heterozygous familial hypercholesterolemia and those at higher CV risk. However, the long-term CV outcomes remain uncertain, with no clear evidence of improvement in major events for alirocumab. Ongoing studies are essential to assess the long-term safety and effectiveness of these treatments, especially regarding total mortality and CV event reduction.

## References

[1] van den Heuvel TWP, Cohen AF, Rissmann R (2016). European drug market entries 2015 with new mechanisms of action. Clin Med.

[2] Trankle CR, Wohlford G, Buckley LF (2019). Alirocumab in acute myocardial infarction: results from the Virginia Commonwealth University alirocumab response trial (VCU-AlirocRT). J Cardiovasc Pharmacol.

[3] Cho KH, Hong YJ (2020). Proprotein convertase subtilisin/kexin type 9 inhibition in cardiovascular disease: current status and future perspectives. Korean J Intern Med.

[4] Al-Mohaissen MA, Ignaszewski MJ, Frohlich J, Ignaszewski AP (2016). Statin-associated muscle adverse events: update for clinicians. Sultan Qaboos Univ Med J.

[5] Glavinovic T, Thanassoulis G, de Graaf J, Couture P, Hegele RA, Sniderman AD (2022). Physiological bases for the superiority of apolipoprotein B over low-density lipoprotein cholesterol and non-high-density lipoprotein cholesterol as a marker of cardiovascular risk. J Am Heart Assoc.

[6] Ruscica M, Corsini A, Ferri N (2020). Clinical approach to the inflammatory etiology of cardiovascular diseases. Pharmacol Res.

[7] Latimer J, Batty JA, Neely RD, Kunadian V (2016). PCSK9 inhibitors in the prevention of cardiovascular disease. J Thromb Thrombolysis.

[8] Benjamin EJ, Blaha MJ, Chiuve SE (2017). Heart disease and stroke statistics—2017 update: a report from the American Heart Association. Circulation.

[9] Page MJ, McKenzie JE, Bossuyt PM (2021). The PRISMA 2020 statement: an updated guideline for reporting systematic reviews. BMJ.

[10] Schmidt AF, Pearce LS, Wilkins JT, Overington JP, Hingorani AD, Casas JP (2017). PCSK9 monoclonal antibodies for the primary and secondary prevention of cardiovascular disease. Cochrane Database Syst Rev.

[11] Ciccarelli G, D'Elia S, De Paulis M, Golino P, Cimmino G (2018). Lipid target in very high-risk cardiovascular patients: lesson from PCSK9 monoclonal antibodies. Diseases.

[12] Khatib R, Khan M, Barrowcliff A, Ikongo E, Burton C, Mansfield M, Hall A (2022). Innovative, centralised, multidisciplinary medicines optimisation clinic for PCSK9 inhibitors. Open Heart.

[13] Sabatine MS, Giugliano RP, Keech AC (2017). Evolocumab and clinical outcomes in patients with cardiovascular disease. N Engl J Med.

[14] Steg PG, Szarek M, Bhatt DL (2019). Effect of alirocumab on mortality after acute coronary syndromes. Circulation.

[15] Schwartz GG, Steg PG, Szarek M (2020). Peripheral artery disease and venous thromboembolic events after acute coronary syndrome. Circulation.

[16] Koskinas KC, Mach F, Räber L (2021). Lipid-lowering therapy and percutaneous coronary interventions. EuroIntervention.

[17] Yu D, Liao JK (2021). Emerging views of statin pleiotropy and cholesterol lowering. Cardiovasc Res.

[18] Rossini E, Biscetti F, Rando MM (2022). Statins in high cardiovascular risk patients: do comorbidities and characteristics matter?. Int J Mol Sci.

[19] Kowara M, Cudnoch-Jedrzejewska A (2021). Different approaches in therapy aiming to stabilize an unstable atherosclerotic plaque. Int J Mol Sci.

[20] Krähenbühl S, Pavik-Mezzour I, von Eckardstein A (2016). Unmet needs in LDL-C lowering: when statins won’t do. Drugs.

[21] Cannon CP, Blazing MA, Giugliano RP (2015). Ezetimibe added to statin therapy after acute coronary syndromes. N Engl J Med.

[22] Jakob T, Nordmann AJ, Schandelmaier S, Ferreira-González I, Briel M (2016). Fibrates for primary prevention of cardiovascular disease events. Cochrane Database Syst Rev.

[23] Schandelmaier S, Briel M, Saccilotto R, Olu KK, Arpagaus A, Hemkens LG, Nordmann AJ (2017). Niacin for primary and secondary prevention of cardiovascular events. Cochrane Database Syst Rev.

[24] Duprez DA, Handelsman Y, Koren M (2020). Cardiovascular outcomes and proprotein convertase subtilisin/kexin type 9 inhibitors: current data and future prospects. Vasc Health Risk Manag.

[25] Murphy SA, Pedersen TR, Gaciong ZA (2019). Effect of the PCSK9 inhibitor evolocumab on total cardiovascular events in patients with cardiovascular disease: a prespecified analysis from the FOURIER trial. JAMA Cardiol.

[26] Bonaca MP, Nault P, Giugliano RP (2018). Low-density lipoprotein cholesterol lowering with evolocumab and outcomes in patients with peripheral artery disease: insights from the Fourier trial (further cardiovascular outcomes research with PCSK9 inhibition in subjects with elevated risk). Circulation.

[27] Schwartz GG, Steg PG, Szarek M (2018). Alirocumab and cardiovascular outcomes after acute coronary syndrome. N Engl J Med.

[28] Sabatine MS, Leiter LA, Wiviott SD (2017). Cardiovascular safety and efficacy of the PCSK9 inhibitor evolocumab in patients with and without diabetes and the effect of evolocumab on glycaemia and risk of new-onset diabetes: a prespecified analysis of the FOURIER randomized controlled trial. Lancet Diabetes Endocrinol.

[29] Nicholls SJ, Puri R, Anderson T (2016). Effect of evolocumab on progression of coronary disease in statin-treated patients: the GLAGOV randomized clinical trial. JAMA.

[30] Nicholls SJ, Kataoka Y, Nissen SE (2022). Effect of evolocumab on coronary plaque phenotype and burden in statin-treated patients following myocardial infarction. JACC Cardiovasc Imaging.

[31] Räber L, Ueki Y, Otsuka T (2022). Effect of alirocumab added to high-intensity statin therapy on coronary atherosclerosis in patients with acute myocardial infarction: the PACMAN-AMI randomized clinical trial. JAMA.

[32] Ray KK, Wright RS, Kallend D (2020). Two phase 3 trials of inclisiran in patients with elevated LDL cholesterol. N Engl J Med.

[33] Stiekema LC, Stroes ES, Verweij SL (2019). Persistent arterial wall inflammation in patients with elevated lipoprotein(a) despite strong low-density lipoprotein cholesterol reduction by proprotein convertase subtilisin/kexin type 9 antibody treatment. Eur Heart J.

[34] Bays H, Gaudet D, Weiss R (2015). Alirocumab as add-on to atorvastatin versus other lipid treatment strategies: ODYSSEY OPTIONS I randomized trial. J Clin Endocrinol Metab.

[35] Wu L, Kong Q, Huang H (2023). Effect of PCSK9 inhibition in combination with statin therapy on intracranial atherosclerotic stenosis: a high-resolution MRI study. Front Aging Neurosci.

[36] Cupido AJ, Kastelein JJ (2020). Inclisiran for the treatment of hypercholesterolaemia: implications and unanswered questions from the ORION trials. Cardiovasc Res.

[37] Bodapati AP, Hanif A, Okafor DK, Katyal G, Kaur G, Ashraf H, Khan S (2023). PCSK-9 inhibitors and cardiovascular outcomes: a systematic review with meta-analysis. Cureus.

[38] Gürgöze MT, Muller-Hansma AH, Schreuder MM, Galema-Boers AM, Boersma E, Roeters van Lennep JE (2019). Adverse events associated with PCSK9 inhibitors: a real-world experience. Clin Pharmacol Ther.

[39] Myers KD, Farboodi N, Mwamburi M (2019). Effect of access to prescribed PCSK9 inhibitors on cardiovascular outcomes. Circ Cardiovasc Qual Outcomes.

[40] Tavori H, Giunzioni I, Fazio S (2015). PCSK9 inhibition to reduce cardiovascular disease risk: recent findings from the biology of PCSK9. Curr Opin Endocrinol Diabetes Obes.

